# Water Limitation Causes Early-Stage Metabolic Perturbation in the Interaction of Soybean and Asian Soybean Rust

**DOI:** 10.1021/acs.jafc.5c07944

**Published:** 2026-01-23

**Authors:** Fernanda R. Castro-Moretti, Gustavo Husein, Eduardo Kiyota, Jessica D. K. Nunes, Giovanna de Carvalho Leite, Silvia A. Lourenço, Claudia B. Monteiro-Vitorello, Lilian Amorim, Paulo Mazzafera

**Affiliations:** † 54538 University of São Paulo, “Luiz de Queiroz” College of Agriculture - ESALQ, Department of Plant Pathology and Nematology, Av. Padua Dias, 11, Piracicaba, São Paulo CEP 13418-260, Brazil; ‡ 54538 University of São Paulo, “Luiz de Queiroz” College of Agriculture - ESALQ, Department of Genetics, Av. Padua Dias, 11, Piracicaba, São Paulo CEP 13418-260, Brazil; § 28132 University of Campinas - Unicamp, Institute of Biology, Department of Plant Biology, R. Monteiro Lobato, 255, Cidade Universitária Zeferino Vaz, Barão Geraldo, Campinas, São Paulo CEP 13083-862, Brazil

**Keywords:** *Phakopsora pachyrhizi*, drought, primary metabolism, secondary metabolism, mass spectrometry

## Abstract

Soybean is a valuable commodity, and its production has been menaced by more frequent drought events and Asian soybean rust, caused by *Phakopsora pachyrhizi*. This study compared the leaf metabolic profile of soybean plants exposed or not to controlled water limitation and then infected with rust at the beginning of fungal colonization. Disease severity was enhanced in plants grown with water limitation, which also had contrasting metabolic profiles compared with those with regular irrigation. Water limitation associated with disease increased defense-related compounds at both 12 and 24 h after inoculation. Naringenin and daidzein, among other flavonoids, accumulated more in inoculated plants, even more so when soybeans were grown in water limitation. Amino acid concentration was negatively correlated with water limitation, which could compromise plant immunity and explain why plants with a stress combination had higher disease severity. The results help us to understand the complex interaction between drought and disease severity, a condition found in the tropics.

## Graphical abstract

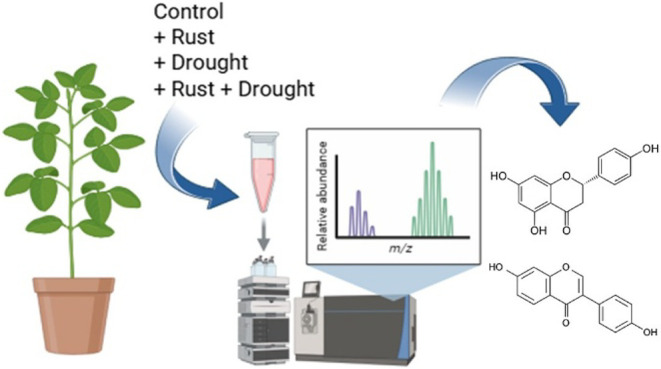

## Introduction

Rust is the most devastating foliar disease of soybean.^1^ It can be caused by two pathogenic agents: *Phakopsora meibomiae*, the causal agent of American rust, and *Phakopsora pachyrhizi*, which causes Asian rust.^2^ American rust causes minor damage to soybean plants, unlike Asian rust, which is aggressive and can cause losses of more than 90%.^3^ Brazil and the United States are the leading soybean producers in the world. Therefore, this disease threatens the global soybean market.^4^ Fungicide application is the main control method for managing Asian soybean rust worldwide.^5^

The increasing demand for resources, driven by a growing population, and the necessity for sustainable agricultural practices in response to climate change pose significant challenges for producers. Aggravated abiotic stress, such as prolonged drought, global warming, and more extreme climate events, are some anticipated scenarios due to climate change.^6,7^ Water distribution worldwide is expected to change as the precipitation and evaporation patterns shift.^8,9^ Extreme precipitation will enhance the risk of flooding areas, and less rain will increase prolonged drought periods.^10,11,12^ Both circumstances are devastating for agriculture. Emerging plant pathogens, pest and disease outbreaks, and higher disease incidence are also associated with global warming and climate changes.^13,14,15^

Modern agriculture has evolved to state-of-the-art technology for plant pathology tools in diagnosis, detection, and disease management.^16^ Machine learning and new technologies to detect foliar features associated with stress, drones expanding that information to vast areas, and automation plus IoT (Internet of Things) will facilitate long-range transmission, bringing new faces to agriculture.^17^ Additionally, omics network analysis will provide continuous resources for researchers to discover new mechanisms behind pathogenic infections. Genomics, transcriptomics, proteomics, and metabolomics are valuable tools for deepening our understanding of the complex relationships among plant pathogenic bacteria, fungi, nematodes, viruses, and each of their hosts. Altogether, all of these new technologies may help to fight the consequences of climate change on agriculture.

Plant metabolomics studies the primary and secondary metabolite profiles in plant cells. Due to its high sensitivity and capacity to detect minor perturbations in a biological system, metabolomics is a promising strategy in plant-pathogen interaction studies.^18^ For the Asian soybean rust pathosystem, studies depicting the metabolomic processes involved in this particular interaction are still scarce. An untargeted approach attempted the identification of defense compounds using the platform Global Natural Product Social Molecular Networking (GNPS).^3^ Nonetheless, their samples were collected only once after the disease symptoms were visible. A year later, the same group unveiled the metabolic profiles of inoculated and noninoculated resistant plants, showing that flavonoids and isoflavonoids were possible defense molecules against the pathogen.^19^ Secondary metabolites such as flavonoids, phenolics, and alkaloids are known for being antimicrobial and are expected to play a role in plant defense against biotic stress.^20^

Few studies correlate rust plant diseases with water stress. A long-term assessment of white pine blister rust caused by *Cronartium ribicola*, also considering drought, showed that disease incidence increased in previously inhospitable areas because of climate conditions that are now more suitable for the disease to happen.^21^ There has yet to be a consensus on the effect of drought on rust severity, revealing the subject’s relevance and the need for more studies. For instance, a long-term assessment of white pine blister rust conducted in white pine and caused by *Cronartium ribicola* showed that disease incidence increased due to climate changes.^21^ The authors discussed how drought-disease interactions varied in intensity or severity and the direction of their relationship (positive or negative) at different elevations. Water limitation reduced disease in low-elevation regions, while increasing disease in higher elevations. Studies with *Puccinia recondita* infecting wheat revealed that rust and drought stresses were additive, as infected plants under water stress had more lesions than diseased plants with regular irrigation.^22^ For *Melampsora apocyni* infecting sword-leaf dogbane (*Apocynum venetum*), the disease was less severe in water-limited treatments. Nonetheless, disease and drought caused more photosynthetic damage than inoculation alone.^23^

Our work aimed to unveil metabolic shifts and molecules involved in the complex interaction between soybean and *P. pachyrhizi* at the early stages of the disease and how water limitation influences plant metabolism and the interaction with the disease. We set up an experiment to compare metabolomic profiles of soybean leaf extracts collected at 0, 12, and 24 h from plants inoculated with rust or mock-inoculated irrigated regularly or with water limitation. The untargeted metabolomics approach using LC–MS revealed metabolites of the interaction in the early stages of the disease when no symptoms were detected. The information described here may contribute to discovering biomarkers and understanding the physiological plant behavior under soybean rust infection. Additionally, the results give biochemical clues for breeding programs to develop more resistant varieties and implement biological control and yet other approaches for efficient and sustainable disease management.

## Material and Methods

All experiments were performed at the Plant Pathology and Nematology Department of the “Luiz de Queiroz” College of Agriculture, University of São Paulo, on the campus of Piracicaba, São Paulo State, Brazil.

### Soil Mixture Preparation

The plants were grown in a mixture containing soil, sand, and manure (2:2:1, v/v/v). The mixture was autoclaved and kept at room temperature for at least 3 days before use. Five 1 L pots were filled with 1 kg of the soil mixture to determine soil saturation capacity, placed in a container with water covering 2/3 of the pots’ external volume, and incubated for 24 h. After this period, the pots had their upper surface sealed with plastic to prevent water loss through transpiration and were placed on a grid to drain excess water. The weight of the saturated soil was recorded when the weight values remained constant (after 48 h). The air-dried soil moisture value and permanent wilting point (PWP) were estimated to determine the gravimetric water content. For air-dried soil, the substrate contained in a 1 L pot was weighed and placed on a tray, weighed, and incubated at room temperature until reaching constant weight (approximately 72 h). The estimation of humidity at the permanent wilting point was carried out following a previously described procedure.^24^ Water limitation treatment was carried out with 65% of the water capacity, while treatment without water limitation was considered to be 80% of the soil water capacity.

### Inoculum Production

Because *P. pachyrhizi* is an obligatory phytopathogen, it was necessary to keep soybean plants inoculated with rust throughout the experimental procedures. To this end, 8 to 10 soybean seeds were sown every week until they reached the V4 stage (four trifoliolate leaves). Irrigation took place daily until the soil was saturated. The plants were fertilized with 6 g of 10-10-5 NPK fertilizer (ICL Fertilizers) when seeded and then were irrigated with 100 mL of a Peters Excel CalMag (ICL Fertilizers) nutrition solution concentrated at 1.5 g L^–1^ every other week after they reached the V2 stage. The plants were inoculated late in the afternoon (at around 7 PM) with a water solution of *P. pachyrhizi* containing 10^5^ spores mL^–1^, in a humid chamber in the dark at 23 °C. Then, the plants were taken to the greenhouse and kept in a plastic tent with a humidifier set to turn on every 2 h. One day before spore collection, the humidifier was turned off to facilitate spore detachment. The spores were collected on the same day the plant material for metabolomics and disease evaluation was inoculated by placing a sheet of paper under the leaves and gently taping them. The collected spores were then transferred to an Eppendorf tube and kept at room temperature until used.

### Plant Material for Metabolomics Analysis

Soybean plants (cultivar Brasmax Lança IPRO) were grown in 1 L pots containing 1 kg of autoclaved substrate (as described before). Fertilization was carried out exactly like the plants kept for the inoculum. The plants were kept in a climate-controlled greenhouse set at 23 °C and 60% humidity. A datalogger (HOBO model MX2301) was placed on the bench to record the daily temperature and average relative humidity. Irrigation was performed daily. To simulate the water deficit conditions, 48 h before inoculation, irrigation was upheld. Previous tests showed that under the greenhouse conditions, this period was enough to drop the soil water content from 80% to approximately 65%, which was defined as the water limitation treatment. Control plants were kept at 80% substrate water content (no water limitation). Further on, the water stress was maintained by individually weighing all pots and adding the amount of water (1 mL = 1 g) to restore 65%. The same procedure was adopted for the control plants. The trial consisted of four treatments (C, control noninoculated and without water limitation; IN, inoculated and without water limitation; WL, noninoculated and with water limitation; INWL, inoculated with water limitation), five biological replicates, and three time points: T0, before inoculation; 12 h after inoculation (HAI); and 24 HAI. These times were defined according to Van de Mortel et al.,^25^ who found that in resistant and susceptible soybean genotypes there were differential gene expression changes within the first 12 h after inoculation.

To confirm the water status of C and WL treatments, three fresh V2-stage trifoliate leaves were collected to perform relative water content analysis, as previously described.^26^ For metabolomics, each biological replicate consisted of one plant. The material collections were carried out in trifoliate V4-stage leaves. Each leaf was immediately frozen in liquid nitrogen after collection and stored at −80 °C. At the end of the test, all collected and frozen leaves were freeze-dried, sealed in plastic bags, and stored airtight with silica at −80 °C until shipped for metabolomic analysis.

### Plant Material for Photosynthesis Evaluation and Disease Severity

A set of plants was kept for nondestructive photosynthesis evaluations, simultaneously with the plants cultivated for metabolomics analysis. They were seeded, fertilized, and had the same four treatments and biological replicates as the plants cultivated for metabolomics. The photosynthesis evaluation time points were T0 (in the morning before inoculation, which was carried out late in the afternoon) and then 36 HAI, which was about 10 AM in the morning. The equipment parameters were defined as 1000 μmol of photons m^–2^ s^–1^ and 400 μmol of CO_2_ m^–2^ s^–1^. Transpiration rate in mmol m^–2^ s^–1^ (E), assimilation rate in μmol m^–2^ s^–1^ (A), intercellular CO_2_ in μmol mol^–1^ (Ci), stomatal conductance to water vapor in mol m^–2^ s^–1^ (Gsw), leaf temperature from energy balance in °C (T) and vapor pressure deficit at leaf temperature in kPa (VPD) were evaluated. Photosynthesis was measured with an infrared gas analyzer (IRGA, LI-COR model LI-6800). Disease severity was measured by estimating the damaged area in the central leaflet of the V3 trifoliate leaf using pictures taken daily after symptoms appeared. Disease severity estimation using photographic records was performed as recommended.^27,28^ The pictures were processed with the software ImageJ (freely available at https://imagej.net/ij) to estimate the damaged area. To ensure that the same area was captured during the photographic evaluations, a mold with a precut circle was placed on the leaves, capturing the same region every time the pictures were taken. A *t*-test was performed to compare the treatments with disease and disease plus water limitation (*p* < 0.05).

### Untargeted Metabolomic Analysis

Metabolite extraction, chromatography runs, and detection were performed by metaSysX GmbH (http://www.metasysx.com). The extraction of primary and secondary metabolites was performed as in Salem et al.,^29^ using 20 mg of dried leaf. The LC–MS was carried out in a Waters ACQUITY Reversed Phase Ultra Performance Liquid Chromatography (RP-UPLC) coupled to a Thermo Fisher Exactive mass spectrometer. C18 columns (100 × 2.1 mm × 1.7 μm) were used for the hydrophilic measurements, and chromatograms were recorded in full-scan MS mode in both positive and negative ionization modes. Data was acquired and processed as in Almanza et al.^30^ using the PeakShaper software. Alignment, filtration, and normalization were completed using in-house software. Feature annotation was performed using an in-house metaSysX database of the chemical compounds. The matching criteria for the polar and nonpolar platforms were 4.1 ppm and 0.098 min deviation from the reference compounds’ mass-to-charge ratio and retention time, respectively.

Partial Least Squares-Discriminant Analysis (PLS-DA) and its most important features, Sparse Partial Least Squares-Discriminant Analysis (sPLS-DA), one-way ANOVA and *t*-tests, volcano plots, relative abundance box plots and heatmaps were performed using the software MetaboAnalyst 5.0.^31^ Data was normalized using log transformation and auto scaling.

### PAL Activity in Soybean Leaves at 12 HAI

Phenylalanine ammonia-lyase (PAL) enzymatic assay was carried out in leaves from soybean plants inoculated with rust or mock-inoculated irrigated regularly or with water limitation collected at 12 HAI. Previously weighted amounts of leaf tissue were extracted and activity analyzed as in Zhang et al.^32^ Protein concentration in the extracts was obtained using a ready-to-use Bradford reagent method (Bio-Rad) for calculation of specific activity.

#### *P. pachyrhizi* Spore Inhibition with Naringenin

Naringenin was added to a *P. pachyrhizi* spore solution to evaluate its effect on germination. A 20 mM naringenin stock solution was prepared with analytical-grade naringenin and ethanol. Naringenin stock solution was diluted and added to spore water solutions to meet approximately 5 × 10^4^ spore mL^–1^. The final concentrations were 1.00, 0.75, 0.50, and 0.25 mM. To eliminate a possible effect of the alcohol, ethanol without naringenin was diluted using the same volumes used to prepare the previous solutions and compared with water. We did not see any negative effect on germination. Thus, water (0 mM) was used as a control in the experiments. For each concentration, four 50 μL drops of the spore solution with naringenin were placed in a disposable Petri dish and incubated in the dark at 23 °C for at least 8 h. Three dishes were used for each concentration (*n* = 3). A small piece of cotton imbibed in distilled water was added inside each Petri dish to simulate a humid chamber. After incubation, the dishes were taken to an optical microscope to count the spores with and without germination tubes. This experiment was repeated three times.

## Results

### Water Limitation Enhances Rust Severity and Changes Photosynthetic Parameters

Water limitation treatment in soybean plants implemented 48 h before inoculation resulted in a significant decrease in transpiration rate, photosynthetic assimilation, CO_2_ concentrations, and stomatal conductance (Supplementary Table S1). The relative water content of control plants (C) averaged 78.8 ± 7.5 and 63.0 ± 7.4 for WL plants, confirming that treatment WL imposed water limitation and restricted plant growth. We evaluated disease severity at 8, 9, 11, 13, 16, and 19 days after inoculation (DAI) on plants grown with and without water limitation. Noninoculated plants showed no symptoms of the disease. Leaf damage caused by rust was more significant in inoculated plants with water limitation (INWL), which was evident at 11 DAI (*p* < 0.5) and even more so afterward (Supplementary Figure S1). Therefore, the combination of abiotic and biotic stresses enhanced the damaged leaf area.

Water limitation treatment in soybean plants implemented 48 h before inoculation resulted in a significant decrease in transpiration rate, photosynthetic assimilation, CO_2_ concentrations, and stomatal conductance (Supplementary Table S1). Less water also led to an increase in leaf temperature and vapor pressure. Nonetheless, 36 h later, similar measurements indicated no significant difference between the treatments with or without water limitation or inoculation. At 0 h after inoculation (HAI), all parameters showed significant differences between treatments with and without water limitation. However, the plants did not show similar differences 36 HAI. The relative water content of control plants (C) averaged 78.8 ± 7.5 and 63.0 ± 7.4 for WL plants, confirming that treatment WL imposed water limitation and restricted plant growth.

### Global Metabolomic Profiling of Soybean Leaf Extracts Resulted in More Than 6 Thousand Features

We carried out LC–MS untargeted metabolomics in soybean leaves from noninoculated and inoculated plants with *P. pachyrhizi*, submitted or not to water limitation. The leaves were collected at 0, 12, and 24 HAI. Nearly seven thousand (6954) features were detected (Supplementary Table S2) and 455 were putatively annotated (Supplementary Table S3). One-way ANOVA analysis revealed 2321 significant features in at least one comparison. Sparse partial least-squares discriminant analysis (sPLS-DA) showed that the clusters were segregated according to time and treatment (Figure 1). The metabolic profile of the treatments before inoculation differed from all others, as the clusters for noninoculated without and with water limitation (C and WL, respectively) at 0 HAI are separated from the remaining clusters (Supplementary Figure S2). The clusters representing the profiles at 12 and 24 HAI are closer, representing similar metabolic profiles. However, it is possible to distinguish between the clusters, meaning that there are significant differences in metabolic composition.

**Figure 1:**
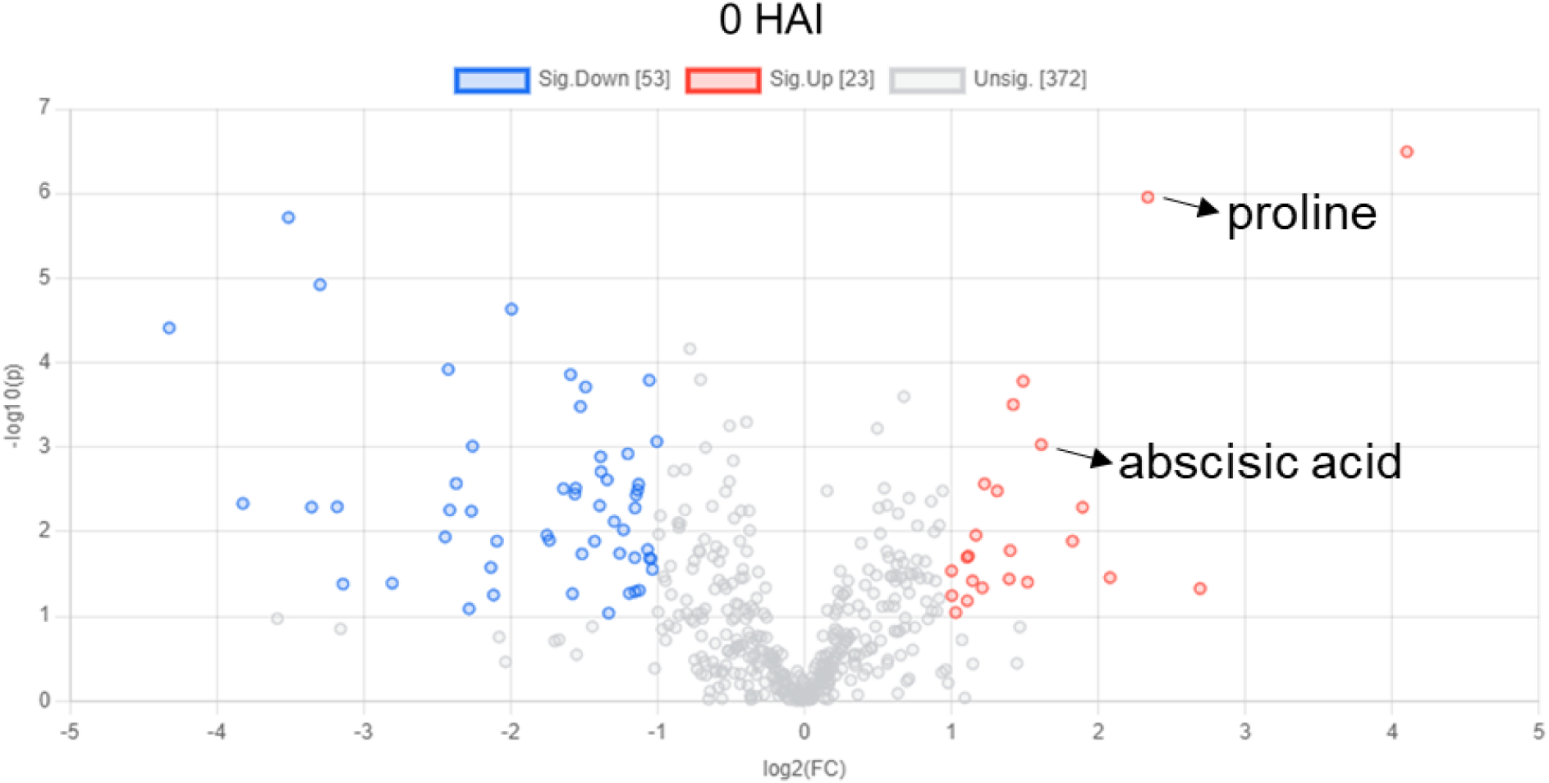
Volcano plot distinguishing more abundant (red) and less abundant (blue) annotated transitions in water-limited soybean leaf extracts noninoculated with rust. Proline and abscisic acid are indicated. Red represents more abundance in the WL, while blue represents less abundance in the WL compared to C at 0 HAI.

### Water Limitation Drives Abscisic Acid and Proline Accumulation in Soybean Plants

At 0 HAI, 1019 transitions significantly differed between WL and C by *t*-test (*p* < 0.05). A volcano plot of the annotated features indicated that 53 features were less abundant when water was limited, and 23 features were relatively more abundant when there was water limitation (Figure 1).

A *t*-test (*p* < 0.05) showed 63 annotated transitions significantly different between C and WL at 0 HAI. The annotated features were classified as amino acids and their derivatives, organic acids, and polyphenols as flavonoid, coumarin, and isoprenoid compounds (Table 1). Proline, l-prolyl-l-asparagine, 5-methylcytosine, *N*-acetyl-l-aspartic acid, l-aspartyl-l-tryptophan, and *O*-phospho-l-serine were among the amino acids and their derivatives that were relatively more abundant in extracts from leaves collected from plants grown in water limitation (Table 1). Abscisic acid was also more abundant in water-limited leaf extracts (Table 1). Fraxin, a coumarin, was among the annotated polyphenols that were highly accumulated in water-limited plants, as most of the molecules from this category were found to be less abundant in WL leaves (Table 1).

**Table 1:** Annotated Features Significantly Different between Water Limitation (WL) and Control (C) before Inoculation (at 0 HAI), Their Synonym or Class, Their Higher or Lower Relative Abundance in WL Compared to C ([WL]), and Their False Discovery Rate Adjusted p-Value (FDR)

Annotated features	Synonym/Class	[WL]	FDR
(2E,4E)-5-[(1S)-1-hydroxy-2,6,6-trimethyl-4-oxocyclohex-2-en-1-yl]-3-methylpenta-2,4-dienoic acid	Abscisic acid/Hormone	Higher	0.019786
3-(Carboxymethyl)-1-(β-d-glucopyranosyl)-1H-indole	Alkaloid	Higher	0.039838
l-Proline	Amino acid	Higher	0.000249
l-Prolyl-l-asparagine	Amino acid derivative	Higher	0.000144
Inosine	Amino acid derivative	Lower	0.002094
2′,3′-Cyclic Cytidine monophosphate	Amino acid derivative	Lower	0.006202
*N*-Acetyl-l-ornithine	Amino acid derivative	Lower	0.006202
Pyroglutamic acid	Amino acid derivative	Lower	0.006202
5-Methylcytosine	Amino acid derivative	Higher	0.013942
l-Kynurenine	Amino acid derivative	Lower	0.019296
l-Tyrosyl-l-proline	Amino acid derivative	Lower	0.019786
4-Pyridoxic acid	Amino acid derivative	Lower	0.02494
Porphobilinogen	Amino acid derivative	Lower	0.030177
β-Hydroxynorvaline	Amino acid derivative	Lower	0.035876
l-Valyl-l-aspartic acid	Amino acid derivative	Lower	0.035876
2′,3′-Cyclic Adenosine monophosphate	Amino acid derivative	Lower	0.037688
l-Threonyl-l-aspartic acid	Amino acid derivative	Higher	0.04254
*N*-Acetyl-l-aspartic acid	Amino acid derivative	Higher	0.042781
l-Aspartyl-l-tryptophan	Amino acid derivative	Higher	0.042781
Propargylglycine	Amino acid derivative	Lower	0.042781
Nicotinamide adenine dinucleotide	Amino acid derivative	Lower	0.042781
*O*-Phospho-l-serine	Amino acid derivative	Higher	0.044772
l-Aspartyl-l-aspartic acid	Amino acid derivative	Lower	0.049261
Benzyl β-d-glucopyranoside	Benzene glycoside	Lower	0.035876
(2S,3S,4S,5R,6R)-6-(3-benzoyloxy-2-hydroxypropoxy)-3,4,5-trihydroxyoxane-2-carboxylic acid	Benzoic acid derivative	Lower	0.035876
3-hydroxy-3-methyl-5-oxo-5-[[(2R,3S,4S,5R,6S)-3,4,5-trihydroxy-6-(2-methyl-4-oxopyran-3-yl)oxyoxan-2-yl]methoxy]pentanoic acid	Chamaemeloside/flavonoid glycoside	Lower	0.009295
(2R,3S,4S,5R,6S)-2-[[(2R,3R,4R)-3,4-dihydroxy-4-(hydroxymethyl)oxolan-2-yl]oxymethyl]-6-[4-hydroxy-3-(3-methylbut-2-enyl)phenoxy]oxane-3,4,5-triol	Cinnamic acid derivative	Higher	0.014187
2-(β-d-glucosyloxy)-cis-cinmic acid	Cinnamic acid derivative	Higher	0.042781
1S,3R,4R,5R)-1,3,4-trihydroxy-5-[(E)-3-(4-hydroxyphenyl)prop-2-enoyl]oxycyclohexane-1-carboxylic acid	Cinnamic acid derivative	Lower	0.042781
[(1aS,1bS,2S,5aR,6S,6aS)-1a-(hydroxymethyl)-2-[(2S,3R,4S,5S,6R)-3,4,5-trihydroxy-6-(hydroxymethyl)oxan-2-yl]oxy-2,5a,6,6a-tetrahydro-1bH-oxireno,[5,6]cyclopenta[1,3-c]pyran-6-yl] 3,4-dihydroxybenzoate	Cinnamic acid derivative	Lower	0.042781
Fraxin	Coumarin glycosides	Higher	0.008104
Sucrose	Dihexose	Higher	0.035876
(2Z,4E)-5-[(1R,3R,5R,8S)-3,8-Dihydroxy-1,5-dimethyl-6-oxabicyclo[3.2.1]oct-8-yl]-3-methyl-2,4-pentadienoic acid	Dihydrophaseic acid/Isoprenoid	Higher	0.009295
Butyl α-d-mannopyranoside	Disaccharides	Lower	0.006703
1-(4-hydroxyphenyl)-3-[(2R,3R,4S,5S,6R)-3,4,5-trihydroxy-6-(hydroxymethyl)oxan-2-yl]oxypropan-1-one	Fatty acyl glycosides	Higher	0.035876
3′,4′,7-Trihydroxyflavone	Flavone	Lower	0.019786
1,5-Anhydro-1-[5,7-dihydroxy-2-(4-hydroxyphenyl)-4-oxo-4H-chromen-8-yl]-2-O-hexopyranosylhexitol	Flavone	Lower	0.042781
3-[4,5-dihydroxy-3-[(2R,3R,4R,5R,6S)-3,4,5-trihydroxy-6-methyloxan-2-yl]oxy-6-[[(2R,3R,4R,5R,6S)-3,4,5-trihydroxy-6-methyloxan-2-yl]oxymethyl]oxan-2-yl]oxy-5,7-dihydroxy-2-(4-hydroxyphenyl)chromen-4-one	Flavonoid	Lower	0.042781
Ramnazin-3-O-rutinoside	Flavonoid glycoside	Lower	0.046905
3-[2-(β-d-Glucopyranosyloxy)-4-methoxyphenyl]propanoic acid	Glycosyl compound	Lower	0.023332
[(1S,4aS,5R,7S,7aS)-4a,5-dihydroxy-7-methyl-1-[(2S,3R,4S,5S,6R)-3,4,5-trihydroxy-6-(hydroxymethyl)oxan-2-yl]oxy-1,5,6,7a-tetrahydrocyclopenta[c]pyran-7-yl] (E)-3-phenylprop-2-enoate	Isoprenoid	Lower	0.002911
Secologanin	Isoprenoid	Lower	0.042781
γ-Valerolactam	Lactone	Lower	0.030177
(d-Glycero-α-d-Manno-Heptopyranosyl)-Dihydrogen phosphate	Lipopolysaccharide	Lower	0.001346
*N*-Acetyl-d-galactosamine|*N*-Acetyl-d-glucosamine|*N*-Acetyl-d-mannosamine	Monosaccharide	Lower	0.022389
2-Oxoadipic acid	Organic acid	Higher	0.006202
d-Galactonic acid	Organic acid	Lower	0.013346
*N*-Methylnicotinic acid	Organic acid	Lower	0.035876
Shikimic acid	Organic acid	Higher	0.035876
Niacin	Organic acid	Higher	0.035876
d-Citramalic acid	Organic acid	Lower	0.035876
*N*-Acetylglutamic acid	Organic acid	Lower	0.037365
β-Hydroxypyruvic acid	Organic acid	Lower	0.042781
Nicotinamide	Organic compound	Lower	0.004402
Arbutin	Phenolic glycosides	Lower	0.042781
α-d-Glucose 1-phosphate|α-d-Galactose 1-phosphate|α-d-Glucose 6-phosphate|α-d-Mannose 1-phosphate	Phosphorylated sugar	Lower	0.006202
d-Ribose 1-phosphate|d-Ribose 5-phosphate	Phosphorylated sugar	Lower	0.042781
*N*-(5-acetamidopentyl)acetamide	Polyamine	Lower	0.000288
Cytidine 3′-phosphate	Ribonucleotide	Lower	0.042781
3-(6-methoxy-1,3-benzodioxol-5-yl)-7-[(2S,3R,4S,5S,6R)-3,4,5-trihydroxy-6-(hydroxymethyl)oxan-2-yl]oxychromen-4-one	Rothindin/Isoflavonoid	Higher	0.035876
(2R,3S,4S,5R,6R)-2-(hydroxymethyl)-6-[[(2R,3S,4S,5R,6S)-3,4,5-trihydroxy-6-(2-hydroxy-4-prop-2-enylphenoxy)oxan-2-yl]methoxy]oxane-3,4,5-triol	Saccharide	Higher	0.035876
2-oct-1-en-3-yloxy-6-[(3,4,5-trihydroxyoxan-2-yl)oxymethyl]oxane-3,4,5-triol	Terpene	Lower	0.030177
6E)-2,10-Dihydroxy-2,6,10-trimethyl-6,11-dodecadien-3-yl β-d-glucopyranoside	Terpenoid	Lower	0.035876

### Treatments with Water Limitation Have Similar Profiles at 12 HAI

ANOVA revealed 498 transitions significantly different (Fisher’s LSD *p* < 0.05) in at least one comparison at 12 HAI in noninoculated plants without and with water limitation and in inoculated plants without and with water limitation (C, WL, IN, and INWL, respectively). A heatmap shows that profiles from inoculated plants with no water limitation (IN) and with water limitation (INWL) were different (Figure 2). Also, there seemed to be more similarities between WL and INWL than between IN and INWL (Figure 2, sections A and B).

**Figure 2:**
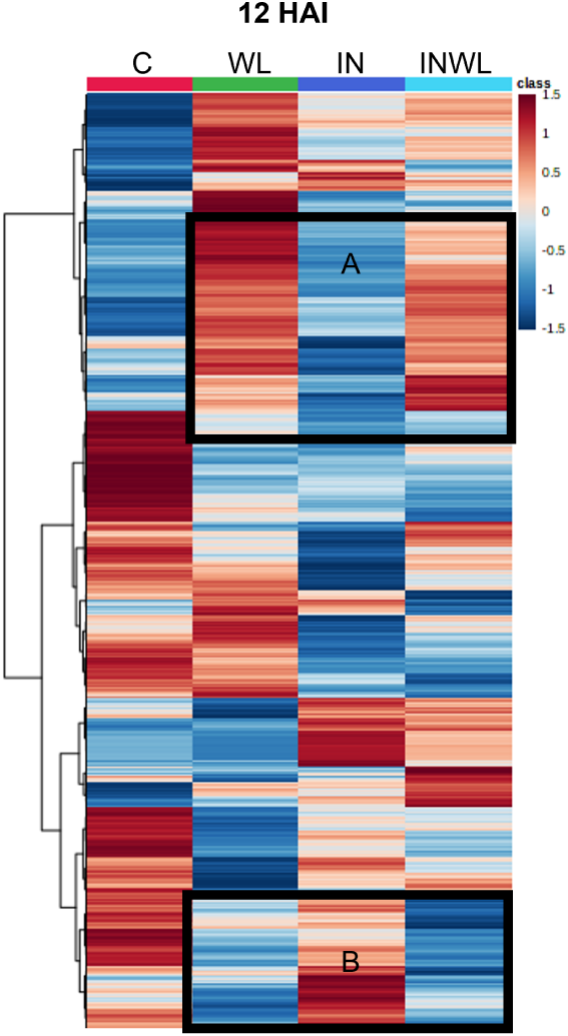
Heatmap displaying comparisons between the metabolic profiles of soybean leaf extracts from noninoculated plants grown with regular irrigation (C), noninoculated plants with water limitation (WL), inoculated plants with regular irrigation (IN), and inoculated plants with water limitation (INWL) at 12 HAI. Sections A and B highlight similarities between WL and INWL. Red represents higher relative abundance, while blue represents lower relative abundance of metabolites.

### Polyphenols Increase with *P. pachyrhizi* Infection at 12 HAI

Twenty-six annotated metabolites were significantly differentiated in at least one comparison (Fisher’s LSD, *p* < 0.05) at 12 HAI. PLS-DA most important features showed polyphenols were relatively more abundant in inoculated plants, as daidzein, naringenin, ergothioneine, isoliquiritigenin, E-astringin (ID PN 1193), 3,4-dihydroxyhydrocinnamic acid (ID PN 1280), trimethylaminobutyrate (ID PP 394), dihydrokaempferol, dihydromethysticin, praeroside II (ID PN 1311), a flavone glycoside (ID PN 1329), isovitexin, *O*-acetyl-l-homoserine, neochanin, uridine diphosphate, and l-methionyl-l-valine were more abundant in IN and INWL (Figure 3). PAL activity did not differ among C, WL, IN, and INWL plants at 12 HAI (*p* < 0.05, Fisher’s LSD, Supplementary Figure S3) nor did its immediate products, cinnamic acid and derivatives (Supplementary Table S3).

**Figure 3:**
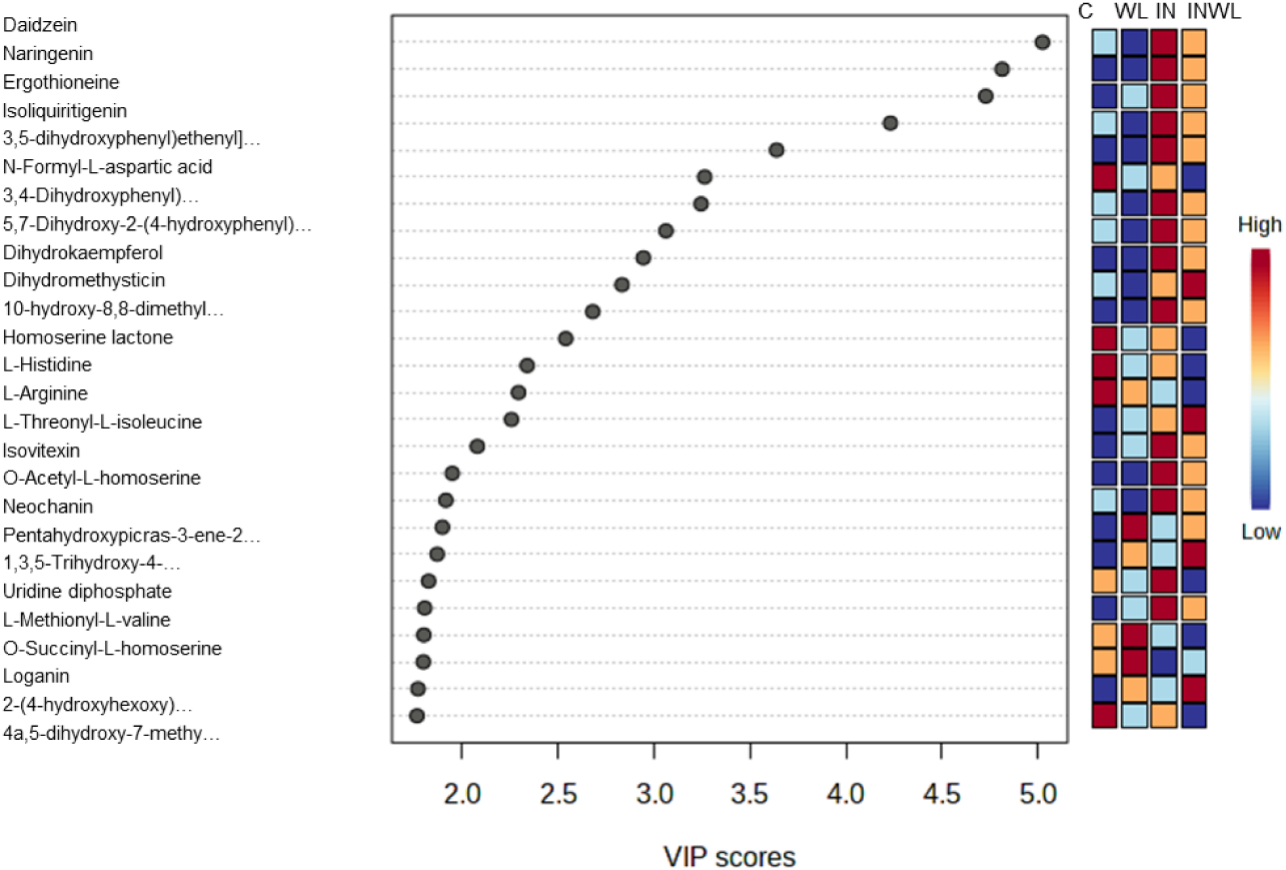
PLS-DA most important annotated features detected in soybean leaf extracts from plants noninoculated and with no water limitation (C), noninoculated and with water limitation (WL), inoculated and with no water limitation (IN), and inoculated and with water limitation (INWL) at 12 HAI. Red represents higher relative abundance, while blue represents lower.

### Inoculated Plants with Water Limitation Have Less Daidzein at 12 HAI

Daidzein is the only annotated feature in the top 30 PLS-DA most significant features at 12 HAI (Figure 4A). It is the one with a higher VIP score when all transitions (annotated and unknowns) are compared (Figure 4A), meaning that it has the most significant influence on profile separation when comparing the four extracts at 12 HAI. Also, it has a higher relative abundance in extracts from inoculated plants that were not subjected to water limitation (Figure 4B). Moreover, daidzein levels in noninoculated plants (C and WL) are significantly lower (*p* < 0.05) than in inoculated plants (IN and INWL; Figure 4B).

**Figure 4:**
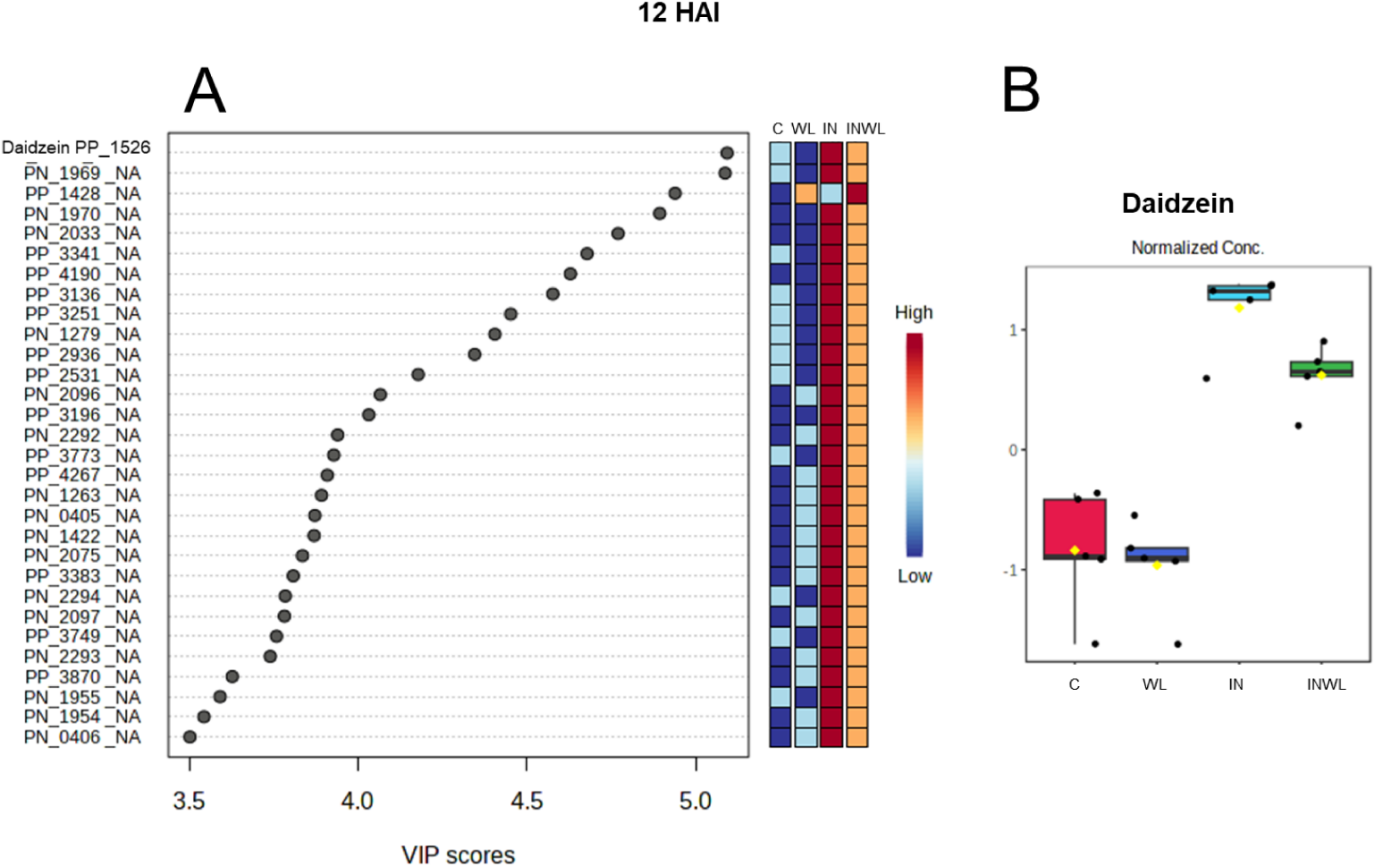
Partial Least Squares-Discriminant Analysis (PLS-DA) most important features (A) ranking features which separate the metabolic profiles of soybean leaf extracts noninoculated and with no water limitation (C), noninoculated and with water limitation (WL), inoculated and with no water limitation (IN), and inoculated and with water limitation (INWL) at 12 HAI. Red represents higher relative abundance, while blue represents lower. Daidzein’s relative abundance in C, WL, IN, and INWL at 12 HAI (B).

### Infected Soybean Metabolic Profile Differences Are Greater at 24 HAI

At 24 HAI, ANOVA showed 670 significantly different features in at least one comparison (*p* < 0.05), which is 172 features more than at 12 HAI. A heatmap shows that the treatments’ profiles differ (Figure 5). Corresponding to 12 HAI, some clusters between WL and INWL are more similar than those between IN and INWL (Figure 5, sections A and B). However, now it is possible to see more clusters in the heatmap where IN and INWL are more similar (Figure 5, sections C and D). At 24 HAI, C is the treatment that differs from all others, as its color pattern in the heatmap is more distinguishable when compared to WL, IN, and INWL (Figure 5, section E).

**Figure 5:**
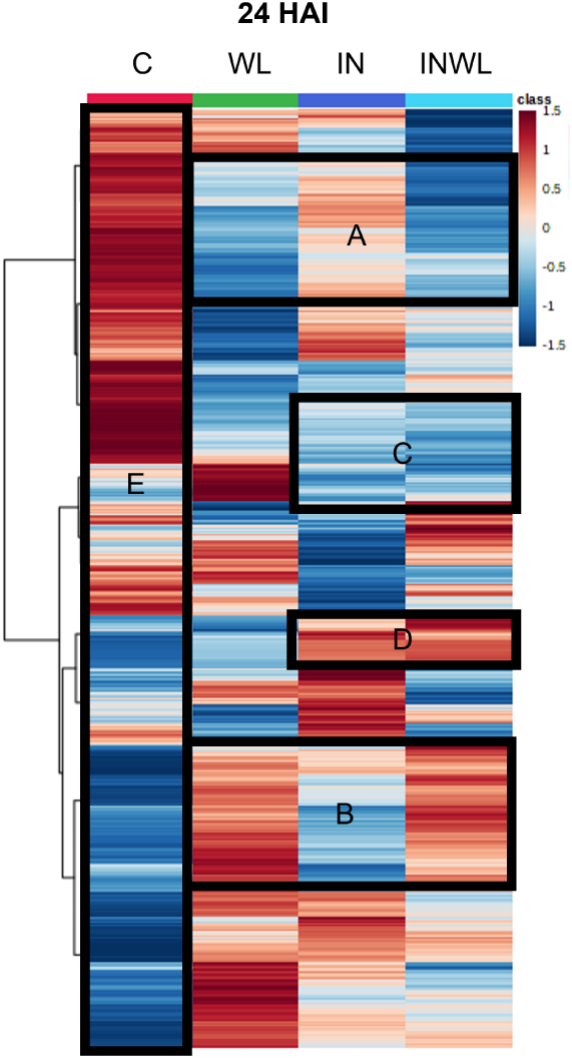
Heatmap displaying comparisons between the metabolic profiles of soybean leaf extracts from noninoculated plants grown with regular irrigation (C), noninoculated plants with water limitation (WL), inoculated plants with regular irrigation (IN), and inoculated plants with water limitation (INWL) at 24 HAI. Sections A and B highlight similarities between WL and INWL. Sections C and D highlight similarities between IN and INWL. Section E highlights how different C is from all other treatments. Red represents higher relative abundance, while blue represents lower relative abundance of metabolites.

### Polyphenols Are Major Separators of Metabolic Profiles between Noninoculated and Inoculated Soybean Plants at 24 HAI

ANOVA of the annotated features identified 33 significant metabolites in at least one comparison at 24 HAI. PLS-DA most important features showed that polyphenols like 3,4-dihydroxycinnamic acid (ID PN 1193), a coumarin (ID PN 1280), isoliquiritigenin, daidzein, junipediol B (ID PN 1034), and praeroside II (ID PN 1311) had high VIP scores, meaning that they were among those responsible for metabolic profile distinction. These molecules were more abundant in IN and INWL (Figure 6).

**Figure 6:**
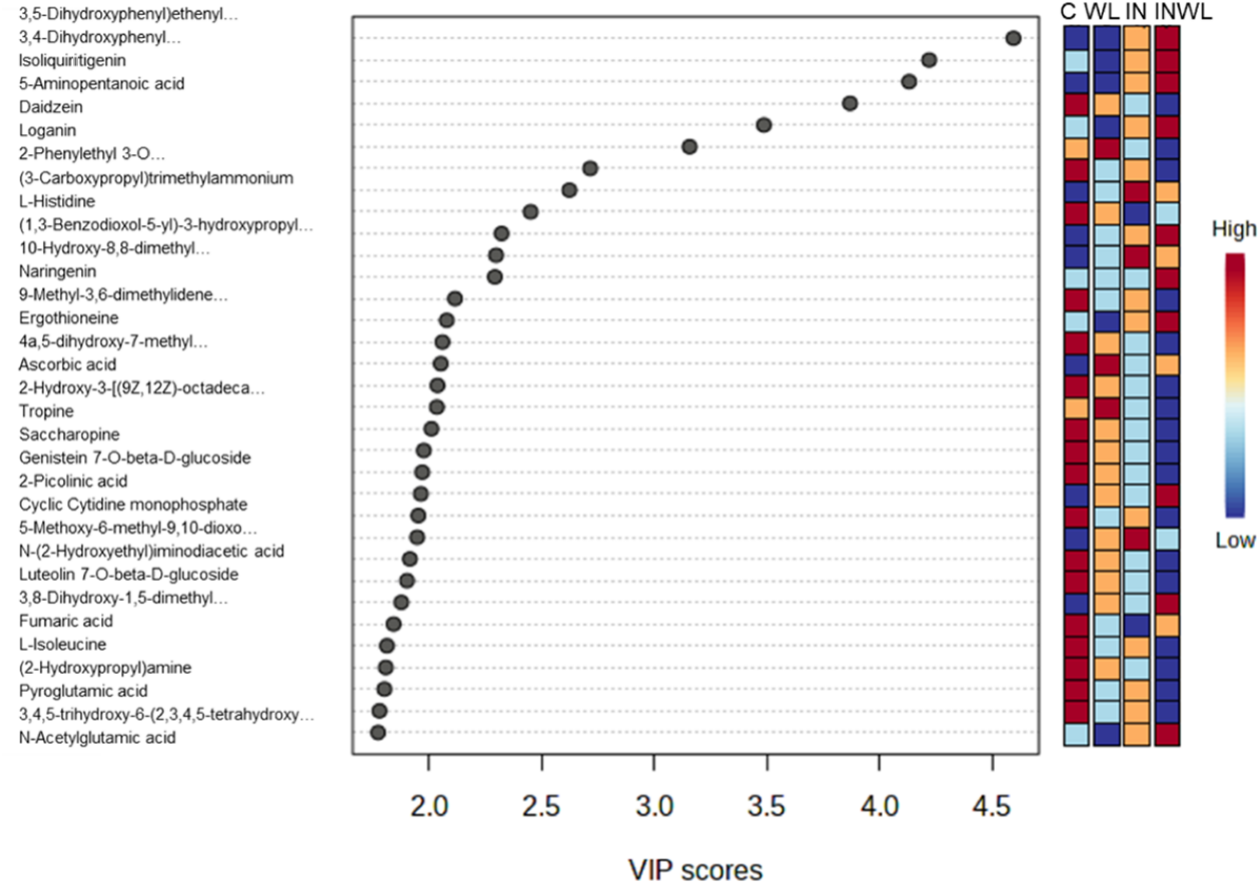
Most important features of Partial Least Squares-Discriminant Analysis (PLS-DA) featuring annotated transitions responsible for metabolic profile differentiation between soybean plants noninoculated with no water limitation (C), noninoculated with water limitation (WL), inoculated with no water limitation (IN), and inoculated with water limitation (INWL) at 24 HAI. Red colors represent higher relative abundance, while blue colors represent lower relative abundance.

### Naringenin Was Only Produced by Rust-Infected Soybean Plants with Water Limitation at 24 HAI

The flavone naringenin was only detected in leaf extracts from soybean rust-inoculated plants grown under water limitation (INWL) at 24 HAI (Figure 7). This was the only annotated transition detected in a single treatment at this collection point.

**Figure 7:**
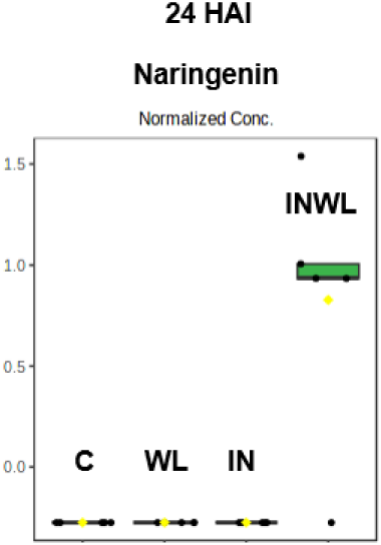
Naringenin relative abundance at 24 HAI in soybean leaf extracts of plants noninoculated with no water limitation (C), noninoculated with water limitation (WL), inoculated with no water limitation (IN), and inoculated with water limitation (INWL).

### Water Limitation Reduces the Relative Abundance of Amino Acids and Their Derivatives in Plants Infected by *P. pachyrhizi*

Top 33 ANOVA-annotated features were plotted in a heatmap (Figure 8). l-serine, l-tryptophan, cytidine 3′-phosphate, *N*,*N*-dimethylglycine, l-leucine, l-isoleucine, afalanine, l-valine, isoleucyl-l-isoleucine, and pyroglutamic acid had lower relative abundance in INWL extracts when compared to IN (Figure 9, sections A and B). l-serine is particularly interesting, as it is also low in control samples C and WL (Figure 8, section C).

**Figure 8:**
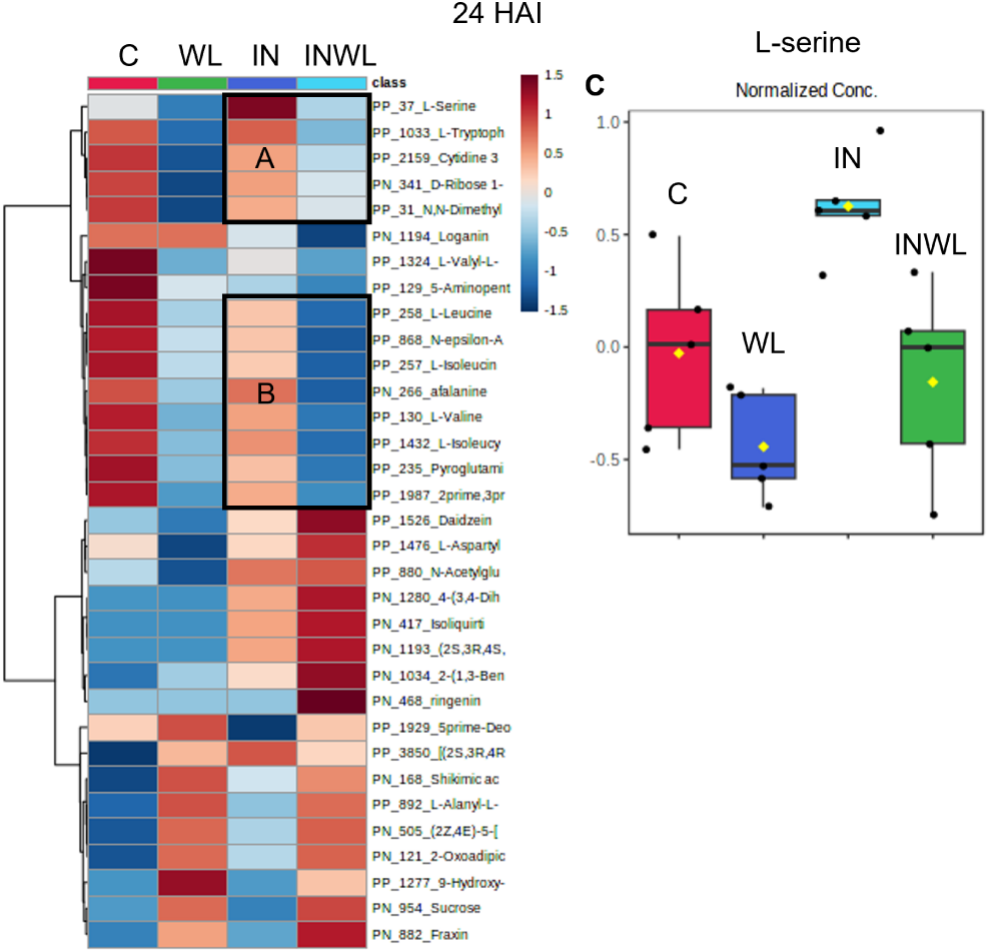
Top 33 annotated features analyzed by ANOVA were plotted in a heatmap. Amino acids and their derivatives with lower relative abundance in INWL than WL (sections A and B). Red colors represent higher relative abundance, while blue represents lower. l-serine relative abundance in C, WL, IN, and INWL extracts (section C).

**Figure 9:**
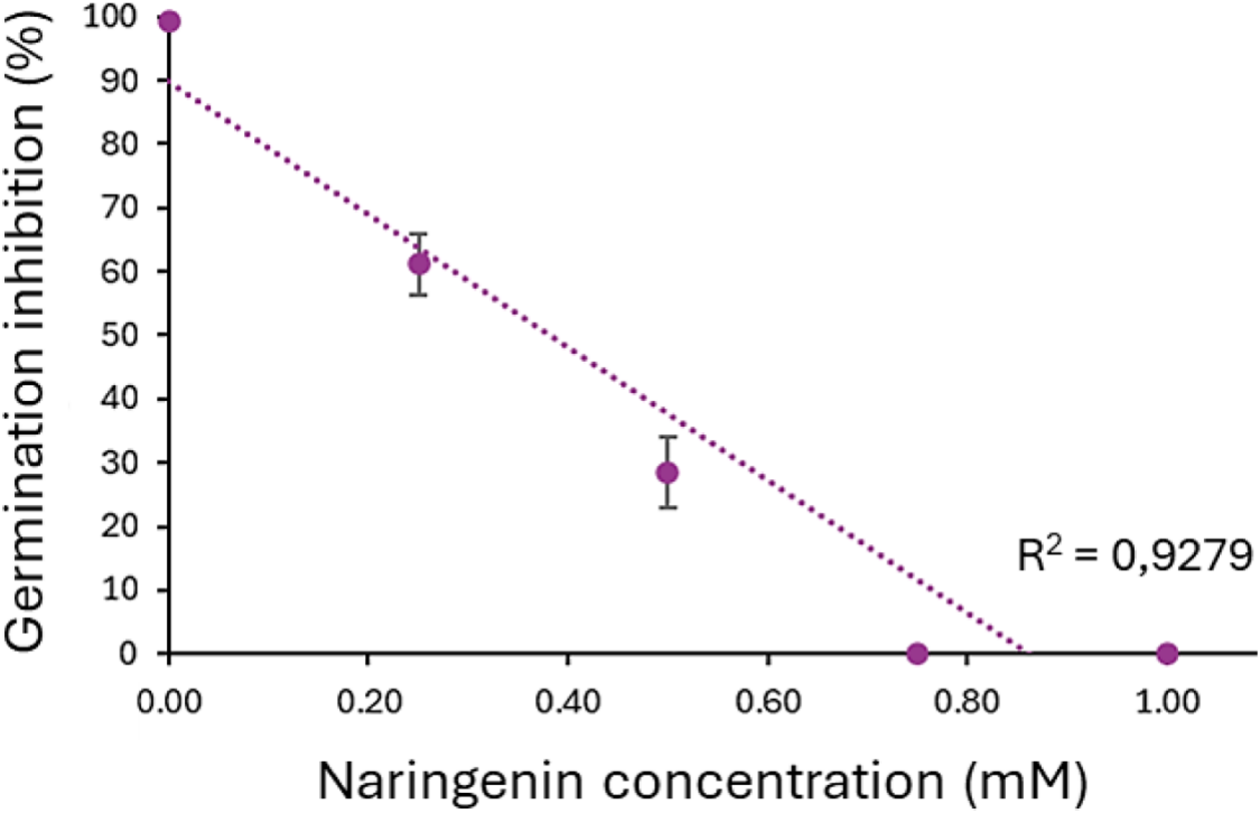
*P. pachyrhizi* spore germination in naringenin solutions.

### Naringenin Inhibits Spore Germination in a Dose–Response Relationship

Germination of *P. pachyrhizi* spores was 99.4% on water. Spore germination was inhibited completely at the concentrations of 1 and 0.75 mM naringenin (Figure 9, Supplementary Table S4).

## Discussion

In this work, we sought to investigate the combined effects of water stress and fungal infection on soybean plants using metabolomics. The time points we analyzed coincide with key stages of the fungal infection process, including appressorium formation, penetration through the cuticle, and invasion and growth of the hyphae into the host tissue.^2,33,34^ However, we extended our experiments for longer periods and demonstrated that prolonged drought increased disease severity from 11 days postinfection (DPI).

Our study detected more than 6000 transitions in soybean leaves inoculated or not with the causal agent of the disease Asian soybean rust, *P. pachyrhizi*, and with or without water limitation. Within this set, we identified 455 transitions putatively annotated, representing the most significant number of detected metabolites in the rust-soybean system described to date. No previous study has found so many transitions nor has identified as many features in an untargeted metabolomics analysis of the interaction between soybean and *P. pachyrhizi* (Supplementary Figure S4A). Additionally, this is the first metabolomics study of the interaction of soybean-ASR and water limitation. Previous studies evaluated soybean primary metabolism,^35,36^ its interaction with rust,^3,19^ or drought,^37–40,41^ but not all factors combined (Supplementary Figure S4B). Studying the metabolomics of plant-microbe interactions is always challenging, as it is difficult to distinguish which metabolites come from plants and which were secreted by microorganisms.^18^ When the infecting agent is bacterial, it is possible to label the pathogen with isotope-labeled compounds added to the culture media before inoculation.^42,43,44^ That technique, however, is possible only with culturable microorganisms, which is not the case with obligate pathogens, like *P. pachyrhizi*. For this particular study, all metabolites were assumed to come from soybean leaves, as the amount of plant tissue present in the early stages of infection is much greater when compared to that of *P. pachyrhizi* and when the dimension of the colonizing pathogen is being considered. Nonetheless, a targeted approach using known standards of metabolites produced only by fungi could be considered in future studies to detect such compounds in the host–pathogen system.

The assay conditions we assessed try to mimic climate change scenarios, wherein some agricultural regions may be affected by prolonged droughts. The phenotypic analysis of leaf damage, the most prominent symptom in the disease’s late stages, showed increased severity when associated with water limitation. While the drought’s additive effect on disease symptoms has not been observed for the disease *in* *A. venetum*,^23^ it has been reported before for rusts in wheat and grapevines.^22,45^

Here, we also observed significant changes in photosynthesis-related physiological parameters only at 0 h after inoculation (HAI) and between control (C) and water-limited (WL) plants. After 36 h, these differences became nonsignificant across all treatments (C, WL, inoculated (IN), and inoculated and water limited (INWL)). In general, plants have photosynthesis reduced at the beginning of plant infection, but the demand for photoassimilates may increase in the infection zone due to plant responses against the pathogen and/or a sink demand represented by pathogen growth, thus restoring or even increasing photosynthesis rates.^46^ However, introducing an abiotic factor such as drought or other environmental changes may lead to nonstandard responses.^21^

As expected, our experiments revealed the significant role of the hormone abscisic acid (ABA) in plants under limited water conditions. ABA is critical in plant development by modulating biochemical signals that trigger stress responses, mainly water stress.^47^ We also detected more sucrose in plants under limited amounts of water. The accumulation of soluble sugars is a common response of plants to water stress, as they diminish the water potential and function as osmolytes, a process known as osmoregulation.^48^ Proline accumulation (Table 1) also reinforces that plants responded to the water limitation by producing osmoprotectants.^49^

The most remarkable features detected were polyphenols in water-limited plants. As reported by other groups, no consensus exists on which molecules are more abundant in experiments under water-limited conditions or rust infection. Various reasons may be related to these discrepancies, such as soybean genotypes, the type of tissue analyzed, stress duration, and the stage of plant development. The instrumentation and database used may also influence the type of detected polyphenols. In our conditions, using leaves in stage 4, at time point 0 HAI, we identified more fraxin, dihydrophaseic acid, rothindin, a cinnamic acid derivative, and an undetermined alkaloid in plants under water limitation. These compounds might be related to protecting cells from oxidative stress,^50,51^ induced by water stress.^52^ Wang et al.^53^ detected in soybean leaves the accumulation of p-dicoumarol, rosmarinic acid, chlorogenic acid B, luteolin-6,8-di-C-glucoside, some alkaloids, and terpenes. In another study, the authors identified increases in total phenolics, particularly caffeic, coumaric, gallic, cinnamic, and ferulic acids.^54^ However, changes in the quantity and quality of phenolic contents have been described for several stresses, but so far, there is no consensus about their effective contribution to scavenging reactive oxygen species (ROS) in stressed plants.^55^ Despite that, the changes we observed here agree with several previous reports, which related changes in phenolic profile with water stress, thus indicating such a condition in our plants.

The infection processes at 12 and 24 HAI led to the most significant changes in metabolite contents. At that time, 12 HAI, the fungus had already invaded the leaf tissues after forming appressorium.^2,33,34^ Additionally, Van de Mortel et al.^25^ verified the most significant changes in the abundance of mRNAs over a 7-day period in soybean genotypes susceptible and resistant to genotypes infected with the Asian rust. These authors observed that several genes related to the biosynthesis of flavonoids, among them phytoalexins, significantly increased the expression in early infection stages in both soybean genotypes. Among these genes were isoflavone synthase and chalcone isomerase, which are responsible for the biosynthesis of daidzein and naringenin, respectively.

PAL activity did not correlate with a higher abundancy of polyphenols at 12 HAI in infected leaves, as it exhibited no significant variation among treatments. Nonetheless, its immediate product (cinnamic acid) also did not differ among treatments at that time point. PAL activity is located at the very beginning of the phenylpropanoid metabolism; thus, we speculate that depending on the position of the metabolite analyzed in the phenylpropanoid metabolism and its concentration in plant tissue, PAL activity may not be altered. Another possibility is that increased naringenin indicates a preferential stimulation of the flavonoid branch, likely via up-regulation of CHS/CHI and/or suppression of competing branches (e.g., lignin).

PAL activity may change during disease evolution, falling to levels even lower than the mock plants.^56^ Also, PAL activity may vary significantly or not depending on the plant genotype, as observed with two black rice cultivars infected with *Xanthomonas oryzae* pv. *oryzae*.^57^ Soybean seedlings infected with *Sclerotinia sclerotiorum* had a large variation of PAL activity, including no significant increase, due to factors such as the specific disease, the soybean cultivar, and the stage of infection.^58^ Olive fruits infected with *Colletotrichum acutatum*, where tolerant cultivars maintain high phenolic pools with only modest PAL changes, have a phenolic profile rather than PAL amplitude correlated with resistance.^59^

We detected various flavonoid classes after rust inoculation with and without a water limitation. Due to their diverse chemical structure and variety, it is believed that they function as relevant components to protect cells against biotic and abiotic stresses and their effects on ROS metabolism.^60^ For instance, daidzein increased in inoculated leaves with or without water limitation. However, the relative abundance of this molecule between the IN and INWL treatments changed over time. Other flavones, such as isoliquiritigenin and naringenin, showed the same trend between IN and INWL at 12 HAI; these metabolites were more abundant in IN, but then at 24 HAI, that relationship was the opposite, being more abundant in INWL, likely reflecting the additive effect of long-term water limitation. Isoliquiritigenin was first identified in soybean root exudate, inducing the activity of the *Bradyrhizobium japonicum* nod genes, more so than inducers such as genistein and daidzein.^61^ This isoflavone is not the most produced by soybeans, but it is the most potent in terms of symbiotic activity, besides protecting the roots of soybeans against *Phytophthora sojae*.^62^ Curiously, even though the biotic stress combined with the water stress reinforces some defense responses, it was not sufficient to impair fungal colonization. Similar studies by Silva et al.^3^ revealed that besides liquiritigenin and daidzein, other flavonoids, such as biochanin A 7-O-d-glucoside, 6-methoxyluteolin-7-rhamnoside, formononetin glucosides, malonyl glycinin, malonyl genistin, and malonyl-daidzein, were detected in abundance in rust-inoculated compared to mock-inoculated plants.

We also highlight the identification of naringenin, which was only detected in INWL at 24 HAI. There are no reports of the naringenin effect produced by soybeans in defense mechanisms or water limitation. However, naringenin has been described as a phytoalexin in other pathosystems.^63–65,66^ In *Arabidopsis*, naringenin induced resistance against *Pseudomonas syringae*, triggering defense responses by upregulating transcription factors for PR genes.^67^ There are also reports on *in silico* models and in vitro assays that predicted the inhibitory effect of naringenin and ABA interaction in plant pathogen enzymatic activity, particularly of *Magnaporthe oryzae* and *Phytophthora infestans*.^68^ In our study, naringenin accumulated in inoculated soybean plants grown with water limitation at later stages, while ABA was more related to noninoculated plants with water limitation at earlier stages. To our knowledge, there are no reports on naringenin’s inhibiting effect on *P. pachyrhizi* infection process. Studies on other soybean pathogens showed that direct application of naringenin inhibited the germination of *Pyricularia* and reduced mycelial growth in *Phytophthora sojae*.^69^ Here we observed that naringenin totally inhibited the germination of soybean rust spores at 0.75 and 1 mM and caused an inhibition of approximately 40% and 70% at 0.25 and 0.5 mM. Also, in tobacco, naringenin induced the accumulation of ROS, a well-known plant defense mechanism.^66^ In addition, other flavonoids like kaempferol, quercetin, and luteolin, also detected here, have proven to be efficient against plant pathogens and pests.^70–72,73^ Further experiments will be carried out to explore the practical use of naringenin and other flavonoids to control soybean rust, focusing on the early infection events and the molecular responses related to induced resistance.

Breeding soybeans for flavonoid accumulation may be a promising strategy to enhance plant resistance against disease when water is limited. Flavone synthesis in soybean leaves and beans has been demonstrated extensively in the literature.^74–76,77^ Plant engineering for naringenin and other flavone accumulation could be an alternative for soybeans, as it is for other crops such as rice and tomato.^78,79,80^ Indeed, the overexpression of chalcone synthase in soybean hairy roots increased the resistance against *Phytophthora sojae*.^62^ The resistance for the same pathogen was also increased in soybean overexpressing an isoflavone reductase.^81^ Wang et al.^60^ reviewed the biosynthesis and metabolic engineering of isoflavonoids in model plants and crops, listing several MYB transcription factors acting as controllers of the expression of chalcone synthase and isoflavone synthase in soybean. In addition to genetically modified plants, other strategies are also promising, such as using nanoparticles to induce flavonoid and other secondary metabolite production.^82,83^

Amino acids play leading roles in primary metabolism. However, they are also important in plant defense and immunity, as they are precursors of polyphenols and hormones and yet can act in ROS scavenging, redox balancing, cytosolic pH buffering, molecular chaperoning, and stabilizing protein structure.^49,84,85^ Our study revealed a lower relative abundance of amino acids in both treatments—rust-infected and noninfected plants—under limited water conditions, which could result in diminished immunity. Another study involving *P. pachyrhizi* showed that amino acid application on infected soybeans reduced disease severity and not only increased phenolic compound accumulation but also lignin and phenylalanine ammonia-lyase activity, a key enzyme to plant defense.^86^

In conclusion, water limitation interfered with the diseased plant’s metabolome. Our study revealed that at our first point after inoculation, 12 HAI, there was a decrease in secondary metabolites in rust-infected plants with water limitations. Curiously, at 24 HAI, that relationship changed, and diseased plants with water limitations had a higher abundance of secondary metabolites. Metabolically, and keeping in mind that most of the changes were related to phenolics, such results indicate that specific compounds are more important in plant response than changes in the total content of this class of metabolites. Our metabolomics analysis provides valuable insights into the interplay between soybean plants, the fungal pathogen *P. pachyrhizi*, and environmental stressors like water limitation. Notably, the observed metabolic shifts likely reflect a multifaceted defense response mounted by the plants upon pathogen recognition, potentially involving Pathogen-Associated Molecular Patterns (PAMPs) and triggering Pathogen-Triggered Immunity (PTI). At the forefront of plant defense mechanisms, PTI represents an early response triggered upon recognizing conserved microbial molecules, such as fungal cell wall components, by plant Pattern Recognition Receptors (PRRs). Although our study does not directly probe the molecular mechanisms underlying PTI, the observed metabolic alterations at critical stages of fungal infection, particularly the significant changes in secondary metabolites like flavonoids, likely signify the activation of defense pathways. The detection of various metabolites, including flavonoids like daidzein, isoliquiritigenin, and naringenin, which are known to play pivotal roles in plant defense against pathogens, underscores the dynamic nature of the plant-pathogen interaction.

These findings hold promise for informing agricultural strategies to enhance crop resilience in the face of evolving environmental challenges. Harnessing the potential of metabolite-driven defense mechanisms, such as flavonoid accumulation, through breeding or biotechnological approaches could empower us to develop resilient crop varieties capable of withstanding pathogen pressures amidst changing climatic conditions.

## Supplementary Material







## Notes

Supplementary Table S2 presents all metabolomics data, nonannotated, of soybean leaf extracts from plants noninoculated with no water limitation (C), noninoculated with water limitation (WL), inoculated with no water limitation (IN), and inoculated with water limitation (INWL) (XLSX)
Supplementary Table S3 presents all metabolomics data, annotated, of soybean leaf extracts from plants noninoculated with no water limitation (C), noninoculated with water limitation (WL), inoculated with no water limitation (IN), and inoculated with water limitation (INWL) (XLSX)
Supplementary Table S1 shows physiological parameters from leaves of plants noninoculated with rust and without water limitation (C), noninoculated with water limitation (WL), inoculated without water limitation (IN), and inoculated with water limitation (INWL) at 0 and 36 h after inoculation (HAI); Supplementary Table S4 presents data of naringenin effect on *Phakopsora pachyrhizi* spore germination; Supplementary Figure S1 shows pictures of rust symptoms at 8, 9, 11, 13, 16, and 19 days after inoculation (DAI) in soybean leaves from plants inoculated with no water limitation (IN) and inoculated with water limitation (INWL); Supplementary Figure S2 is a plot that summarizes the metabolomic profiling performed on all treatments and time points in this study; Supplementary Figure S3 is a graph representing PAL activity and a cinnamic acid relative abundance from soybean leaf extracts obtained from plants noninoculated with rust and with normal irrigation (C), noninoculated with water limitation (WL), inoculated with rust and normal irrigation (IN), and inoculated and with water limitation (INWL), collected at 12 h after inoculation (HAI); Supplementary Figure S4 compiles recent soybean metabolomics studies with *P. pachyrhizi* and water limitation and the number of features obtained by metabolomics analyses in soybean leaves in this study and others from the literature (PDF)

## Abbreviations

LC–MS: liquid chromatography–mass spectrometry
V4: vegetative stage 4
E: transpiration rate in mmol m^–2^ s^–1^
A: assimilation rate in μmol m^–2^ s^–1^
Ci: intercellular CO_2_ in μmol mol^–1^
Gsw: stomatal conductance to water vapor in mol m^–2^ s^–1^
T: leaf temperature from energy balance in °C
VPD: vapor pressure deficit at leaf temperature in kPa
C: control without inoculation and without water limitation
IN: inoculated treatment without water limitation
WL: water limitation control without inoculation
INWL: water limitation and inoculation treatment
T0: time point before inoculation
HAI and DAI: hours and days after inoculation
PAMPs: pathogen-associated molecular patterns
PTI: pathogen-triggered immunity
