# Supplementary material for: Water Limitation Causes Early-Stage Metabolic Perturbation in the Interaction of Soybean and Asian Soybean Rust
Source: J Agric Food Chem. 2026 Jan 23;74(4):4045–58. doi: 10.1021/acs.jafc.5c07944 (PMC12879939; doi:10.1021/acs.jafc.5c07944)
Supplement: Supplementary file 3 [file jf5c07944_si_003.pdf]

Supporting Information

Water limitation causes early-stage metabolic perturbation in the interaction of soybean and Asian soybean rust

Fernanda R. Castro-Moretti[1*]; Gustavo Husein[2]; Eduardo Kiyota[3]; Jessica D.K. Nunes[1]; Giovanna de Carvalho Leite[1]; Silvia A. Lourenço[1]; Claudia B. Monteiro-Vitorello[2]; Lilian Amorim[1]; Paulo Mazzafera[3].

[1]University of São Paulo, “Luiz de Queiroz” College of Agriculture - ESALQ, Department of Plant Pathology and Nematology. Av. Padua Dias, 11. CEP 13418-260, Piracicaba – São Paulo, Brazil.

[2]University of São Paulo, “Luiz de Queiroz” College of Agriculture - ESALQ, Department of Genetics. Av. Padua Dias, 11. CEP 13418-260, Piracicaba – São Paulo, Brazil.

[3]University of Campinas - Unicamp, Institute of Biology, Department of Plant Biology, R. Monteiro Lobato, 255, Cidade Universitária Zeferino Vaz, Barão Geraldo. CEP 13083-862, Campinas – São Paulo, Brazil.

*Corresponding author: Fernanda R. Castro-Moretti email: fmoretti@usp.br

Keywords: *Phakopsora pachyrhizi*, drought, primary metabolism, secondary metabolism, mass spectrometry.

Funding: FAPESP (São Paulo Research Foundation) Grant Number 2019/13191-5

Supplementary Table S1. Transpiration rate (E), assimilation rate (A), intercellular CO_2 (Ci), stomatal conductance to water vapour (Gsw), leaf temperature from energy balance (T) and vapour pressure deficit at leaf temperature (VPD) obtained from leaves of plants non-inoculated with rust and without water limitation (C), non-inoculated with water limitation (WL), inoculated without water limitation (IN) and inoculated with water limitation (INWL) at 0 and 36 h after inoculation (HAI). Averages ± standard deviation are shown; n = 10 at 0 HAI and n = 5 at 36 HAI. Different letters represent significant averages between treatments within the same time-point with $p<0.01$.

treatment	time	E (mmol m^{-2} s^{-1})	A (µmol m^{-2} s^{-1})	Ci (µmol mol^{-1})	Gsw (mol m^{-2} s^{-1})	T (°C)	VPD (kPa)
C	0 HAI	6.95 ± 0.54a	26.5 ± 1.5a	306 ± 8.9a	0.63 ± 0.07a	26.37 ± 0.16a	1.16 ± 0.04a
WL		4.08 ± 1.51b	20.9 ± 2.9b	255 ± 36b	0.31 ± 0.15b	27.14 ± 0.43b	1.35 ± 0.09b
C	36 HAI	5.0 2± 0.95a	23.62 ± 3.03a	277 ± 15a	0.39 ± 0.10a	27.10 ± 0.33a	1.29 ± 0.07a
IN		4.35 ± 1.95a	19.97 ± 6.03a	260 ± 47a	0.32 ± 0.17a	27.29 ± 0.53a	1.36 ± 0.14a
WL		5.57 ± 1.71a	23.6 1± 1.72a	278 ± 33a	0.43 ± 0.18a	27.05 ± 0.60a	1.33 ± 0.07a
INWL		4.23 ± 1.72a	21.39 ± 5.70a	252 ± 40a	0.32 ± 0.16a	27.41 ± 0.37a	1.43 ± 0.11a

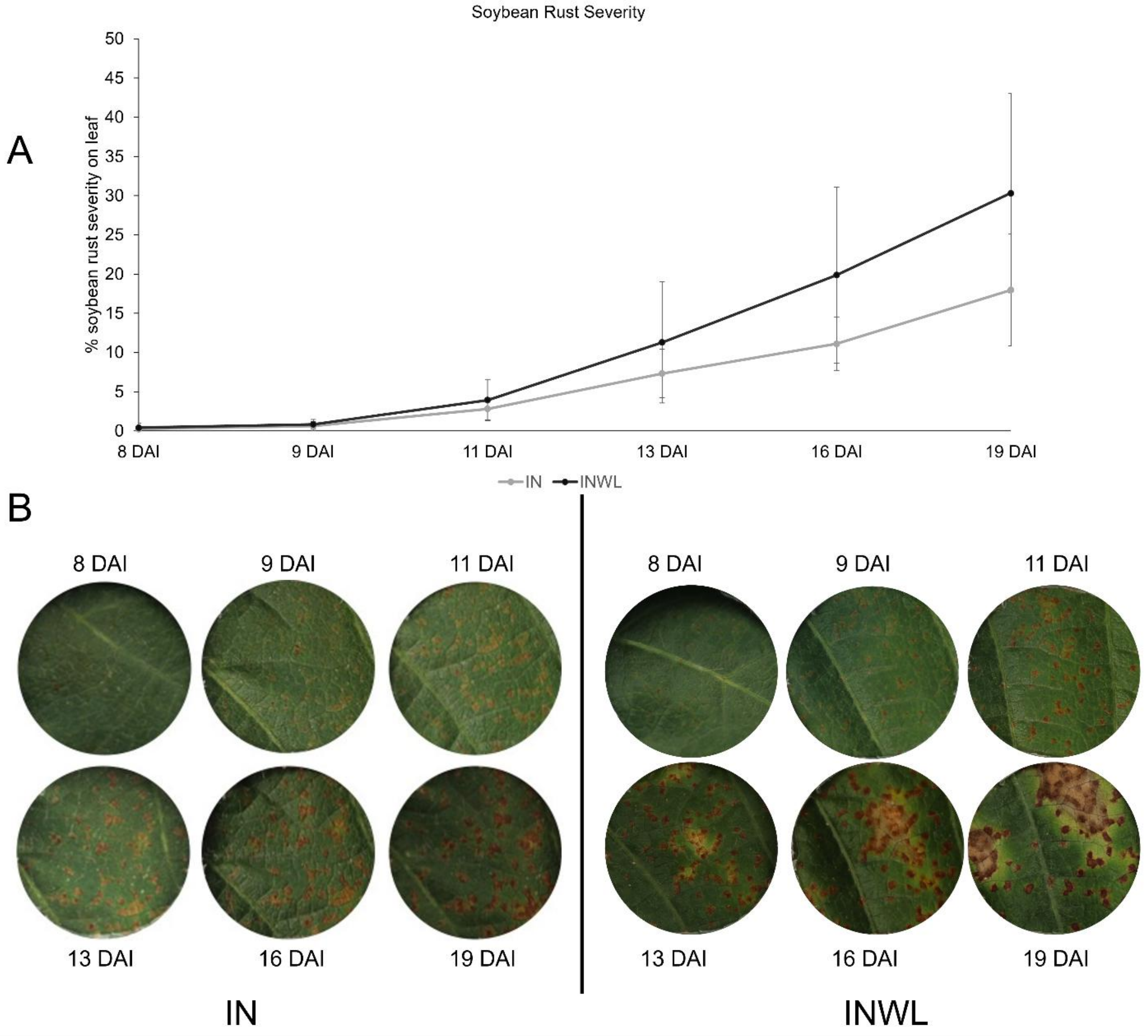

Supplementary Figure S1. Disease severity (A) 8, 9, 11, 13, 16 and 19 days after inoculation (DAI) in soybean leaves from plants inoculated with no water limitation (IN) and inoculated with water limitation (INWL). Diseased areas (B) at 8-19 DAI in leaves from soybean plants inoculated with no water limitation (IN) and inoculated with water limitation (INWL). Non-inoculated plants had no symptoms of the disease. Severity was significantly different between IN and INWL at 11 DAI and later according to Tukey's Test ($p>0.5$).

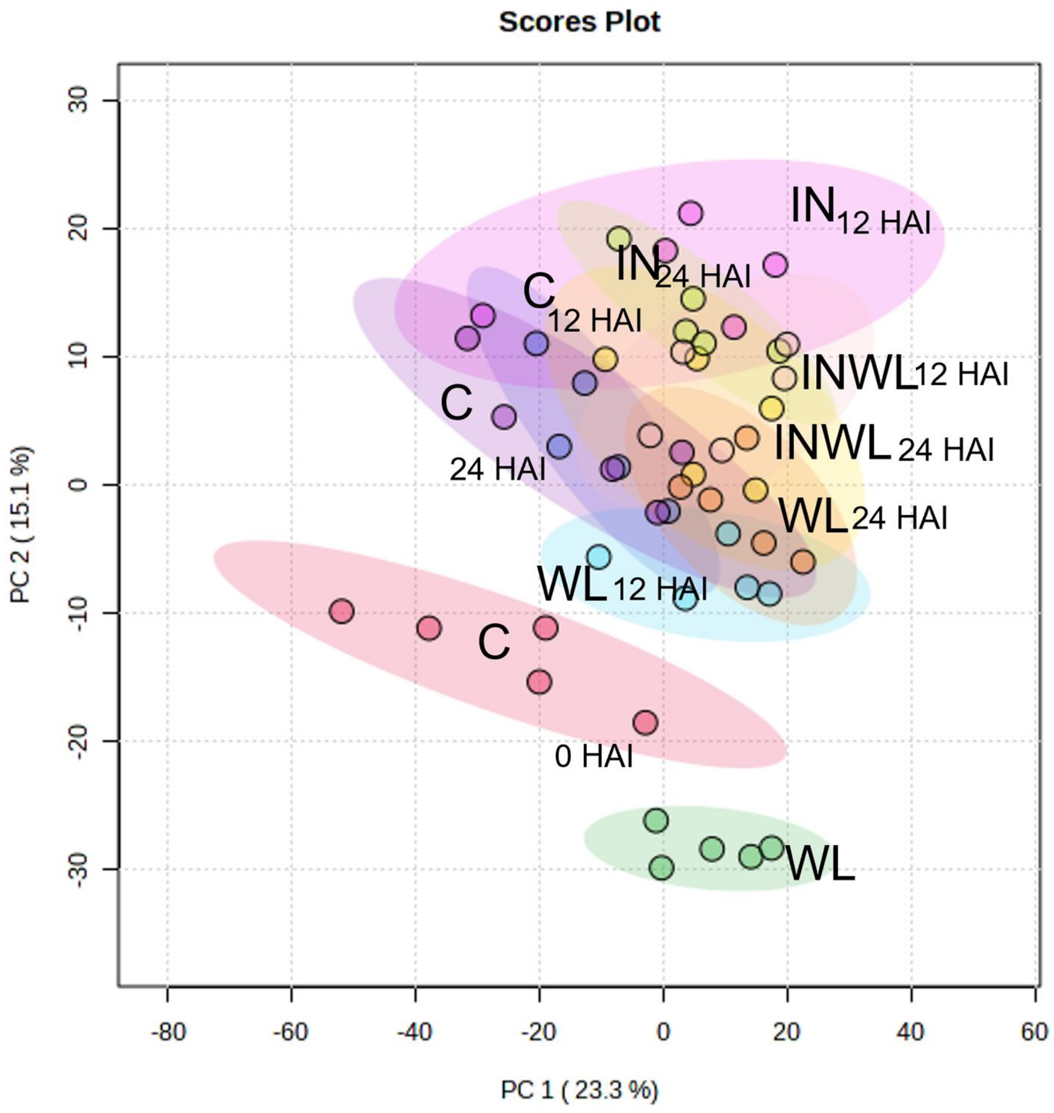

Supplementary Figure S2. Sparse Partial Least Square-Discriminant Analysis (sPLS-DA) of global profiling from soybean leaf extracts obtained from plants non-inoculated with rust and with normal irrigation (C), non-inoculated with water limitation (WL), inoculated with rust and normal irrigation (IN) and inoculated and with water limitation (INWL), collected at 0, 12 and 24 h after inoculation (HAI).

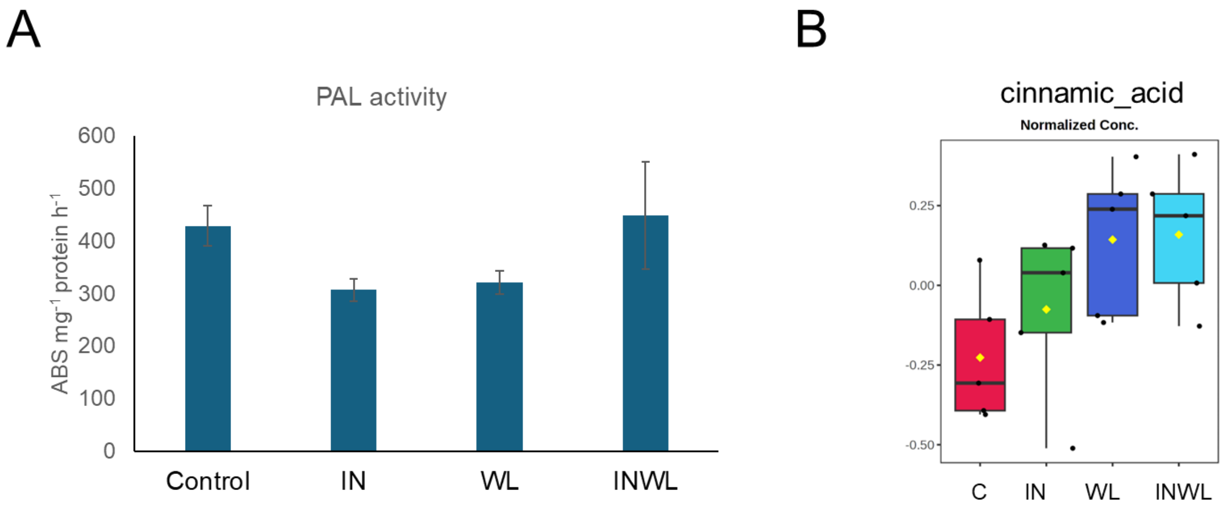

Supplementary Figure S3. PAL activity (A) and a cinnamic acid relative abundance (B) from soybean leaf extracts obtained from plants non-inoculated with rust and with normal irrigation (C), non-inoculated with water limitation (WL), inoculated with rust and normal irrigation (IN) and inoculated and with water limitation (INWL), collected at 12 h after inoculation (HAI) with no significance between treatments according to Fisher´s LSD ($p<0.05$, n=5).

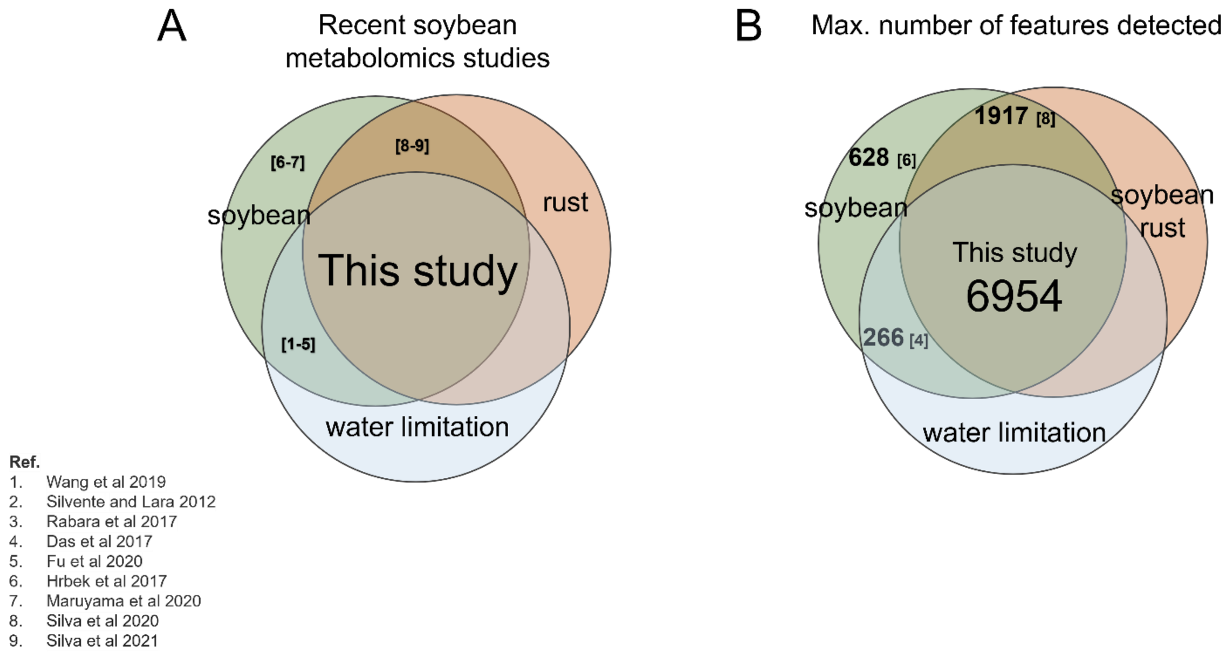

Supplementary Figure S4. Recent soybean metabolomics studies with *P. pachyrhizi* and water limitation (A) and maximum number of features obtained by metabolomics analyses in soybean leaves in this study and others from the literature (B).

(Supplementary Table S2 and S3 as excel file)

Supplementary Table S4. Average spore germination ± standard deviation of spores treated with 1, 0.75, 0.50, 0.25 and 0 mM of naringenin, n=3.

Naringenin concentration (mM)	Average germination (%)
1.00	0.0 ± 0.0
0.75	0.0 ± 0.0
0.50	28.6 ± 7.4
0.25	61.2 ± 9.3
0.00	99.4 ± 0.7
